# Treatment of a recurrent ischial ulcer with injected exosomes

**DOI:** 10.1093/jscr/rjac271

**Published:** 2022-06-28

**Authors:** Genevieve E Messa, Rafael P Tiongco, Frank H Lau

**Affiliations:** Department of Surgery, Louisiana State University Health Sciences Center New Orleans (LSUHSC-NO), New Orleans, LA, USA; Tulane University School of Medicine, New Orleans, LA, USA; Department of Surgery, Louisiana State University Health Sciences Center New Orleans (LSUHSC-NO), New Orleans, LA, USA

## Abstract

Pressure ulcers (PUs) affect 2.5 million patients per year. Even after successful reconstruction, 50% of PUs recur. Patients with multiply recurrent PUs eventually consume all locoregional donor sites. This underscores the need for novel, less invasive approaches in PU reconstruction. Here, we report the first successful use of mesenchymal stem cell exosomes in PU reconstruction. The patient presented with a right ischial ulcer that persisted despite 9 months of wound care and appropriate antibiotic therapy. After six subcutaneous ExoFlo exosome injections over 8 weeks, the PU was completely healed. Additional studies of this promising technology should be performed.

## INTRODUCTION

Pressure ulcers (PUs) affect 2.5 million individuals per year. Treatment costs range from $20 900 to $151 700 per ulcer [1]. Most PUs develop in the sacral, ischial and greater trochanteric regions. The standard of care for Stage 3 and 4 PUs is surgical excision followed by reconstruction with locoregional flaps. However, even successfully reconstructed ulcers have a 50% 1-year recurrence rate [2]. PU patients needing multiple reconstructions can deplete their locoregional donor sites, necessitating free tissue transfer [3]. To reduce the cost and morbidity of PU reconstruction, novel, less invasive reconstructive strategies should be studied.

One such new strategy are mesenchymal stem cell (MSC) exosomes [4]. Exosomes are extracellular vesicles that modify target cell physiology, thereby enhancing wound healing [5]. Multiple rounds of treatment with exosomes are well-tolerated [5, 6]. We present the first case report of successful reconstruction of a recurrent PU using MSC exosomes.

## CASE REPORT

**Figure 1:**
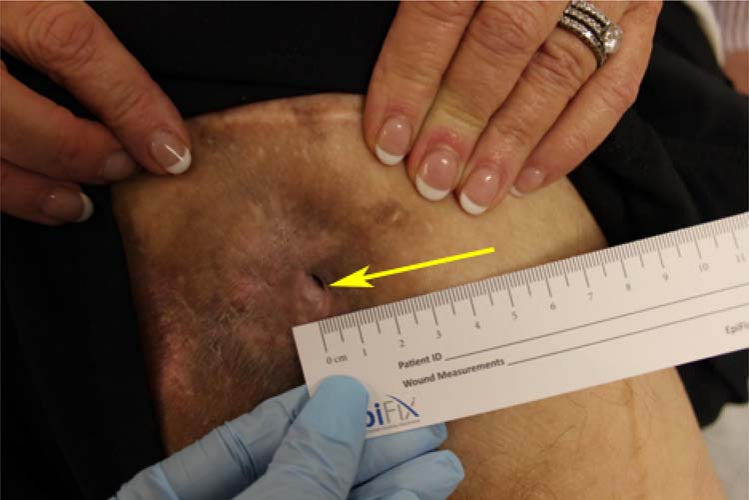
Patient’s right ischial PU on initial encounter. PU has a 4×4 cm, circular scar region surrounding the sinus tract (arrow).

A 38-year-old male with a history of cerebral palsy and multiple PUs requiring over 50 surgeries presented with a recurrent, right ischial ulcer measuring 5x4x5 cm that failed to heal after 9 months of wound care. Despite targeted antibiotic therapy, the ulcer was colonized with methicillin-resistant Staphylococcus aureus (MRSA) (Fig. 1). Several scars were present from previous PU flap reconstructions.

The patient underwent excisional debridement of the ischial PU with immediate reconstruction using a 22x12 cm posterior thigh flap. The procedure was uncomplicated. The patient was kept inpatient with strict bedrest and pressure offloading measures, and on intravenous vancomycin for 1 week. He was discharged home on post-operative day 6 since he had adequate support mechanisms at home.

Two weeks post-procedure, the patient returned with wound dehiscence and decreased blood supply at the tip of the flap. Drainage, but no odor, was noted. Cultures grew *Enterococcus*, which was treated with oral antibiotics and that week, the patient returned to the OR for excisional debridement of the necrotic portion of flap and readvancement of the flap. The surgery was uncomplicated.

**Figure 2:**
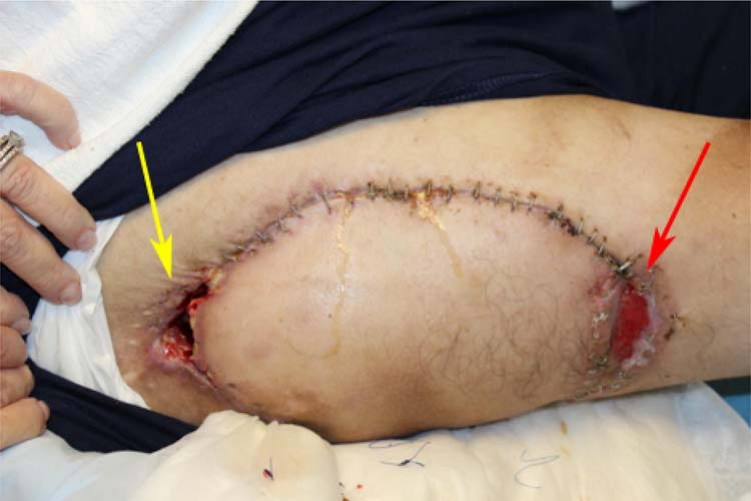
Wound dehiscence after correction of prior wound dehiscence. Sinus tract (yellow arrow) and donor site (red arrow) both dehisced.

Three weeks after the flap readvancement, the patient returned with recurrence of his right ischial ulcer (Fig. 2). Pus was draining but no cellulitis or abscesses were present. Cultures grew pansensitive *Enterobacter*. The infection was managed with 2 weeks of Bactrim PO and packing of the open sinus tract and donor site.

## EXOSOME TREATMENT

After 2 weeks, the wound was clean and showed no signs of infection. Rather than a third surgical revision, the patient was offered and agreed to trial exosomes (ExoFlo, Direct Biologics). One cc of exosomes was diluted in 4 cc of sterile saline injected into the base and walls of the wound.

**Figure 3:**
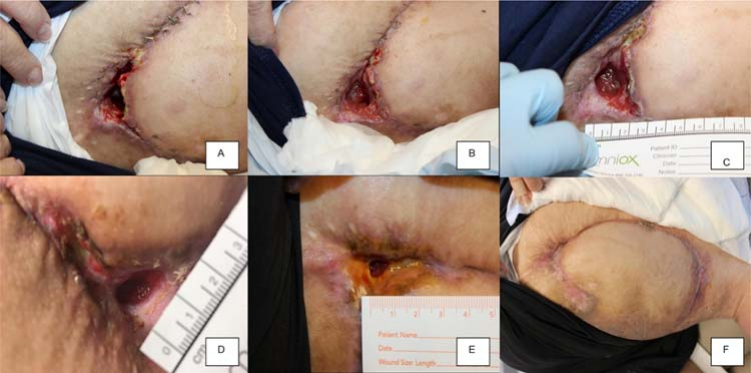
ExoFlo exosome injection course showing progressive improvement in healing. (**A**) 1 week post-injection; (**B**) 2 weeks; (**C**) 3 weeks; (**D**) 5 weeks; (**E**) 6 weeks; (**F**) 8 weeks.

The patient returned the following week with improvement. The base of the wound, which was previously too deep to see, was newly visible (Fig. 3A). No signs of infection were present. A second round of exosomes was administered with continued reduction in the ulcer volume.

Additional rounds of exosomes were administered on the third, fifth, sixth and seventh weeks with marked improvement (Fig. 3B–F). On the eighth week, examination revealed a healed wound. The PU remained healed for 2 years, until the patient returned with a recurrence secondary to prolonged (6+ h) pressure.

## DISCUSSION

Surgical reconstruction of PUs remains the gold standard, but postoperative management is resource intensive, recurrence is common and donor sites are limited. New treatment options that do not require donor sites are indicated, particularly for patients with a history of recurrence or whose prior reconstructions have depleted their locoregional reconstructive options. This case report is the first to show that exosomes are a nonsurgical option for this population.

### Mechanisms of action

Recent studies suggest that exosomes are responsible for regenerative benefits formerly attributed to stem cells [7–9]. Stem cells do not augment wound healing via implantation. Instead, MSCs release paracrine factors, such as exosomes, to stimulate wound healing [7]. Cell-free treatment with exosomes reduces the risk of transferring mutated or damaged DNA, and exosomes may remain in circulation longer as compared with an equivalent dose of infused MSCs [9].

The primary mechanism of exosome action is the horizontal transfer of mRNA, miRNA and proteins. Exosomes contain mRNA, miRNA, pre-miRNA and other noncoding RNA, annexins and tetraspanins (i.e. CD63, CD81, CD9), heat shock proteins, Alix, tsg101 and clathrin [10]. The transfer of these molecules from stem cells to damaged cells promotes wound-healing. For example, exosomes promote reepithelialization by inducing epithelial cell proliferation, angiogenesis and secretion of collagen and elastin [9].

Exosomes may limit immune and inflammatory responses by decreasing the levels of proinflammatory cytokines and chemokines, while stimulating the secretion of IL-10, an anti-inflammatory cytokine [7, 11]. A study by Zhang *et al*. supported the potential immunological activity of exosomes, demonstrating that exosomes induced the secretion of anti-inflammatory cytokines and polarization of T cells into T regulatory cells. The anti-inflammatory response mediated by exosomes was believed to be responsible for improved allogenic skin graft survival [11].

In chronic wounds, exosomes induced normal and diabetic human fibroblast growth [7]. Wound healing transcriptional changes included induction of cell cycle genes (c-myc, cyclin A1, cyclin D2), growth factors (HGF, IGF1, NGF, SDF1) and IL-6 by upregulation of STAT3 (a transcription factor active in wound healing), AKT and ERK1/2 signaling cascades [7]. In addition, a study by Fang *et al*. found that MSC-derived exosomes contain RNAs that inhibit myo-fibroblast formation, thereby limiting scar formation in wound healing [8]. Exosomes may attenuate the fibroblast to myo-fibroblast transition by inhibiting expression of the α-smooth muscle actin gene and reducing collagen deposition at wound sites [8].

## CONCLUSION

We report the first successful of MSC-derived exosomes in PU wound healing without the need for surgical intervention and preventing recurrence. Given their potential as a novel treatment for recurrent PU, additional prospective studies are indicated.

## References

[1] Are we ready for this change? Agency for Healthcare Research and Quality. https://www.ahrq.gov/patient-safety/settings/hospital/resource/pressureulcer/tool/index.html. Published April 2011. 14 February 14 2022, date last accessed.

[2] Vasconez LO , Schneider WJ, Jurkiewicz MJ. Pressure sores. Curr Probl Surg 1977;14:1–62. 10.1016/s0011-3840(77)80007-5 195770

[3] Robertson C , Patterson C, Hilaire HS, Lau FH. Free tissue transfer in pressure ulcer reconstruction: a systematic review. J Reconstr Microsurg Open 2021;06:e35–9.

[4] Rani S , Ritter T. The exosome – a naturally secreted nanoparticle and its application to wound healing. Adv Mater 2016;28:5542–52. 26678528 10.1002/adma.201504009

[5] O’Loughlin A , Woffindale C, Wood M. Exosomes and the emerging field of exosome-based gene therapy. Curr Gene Ther 2012;12:262–74. 22856601 10.2174/156652312802083594

[6] An Y , et al. Exosomes from adipose-derived stem cells and application to skin wound healing. Cell Prolif 2021;54:e12993. 33458899 10.1111/cpr.12993 PMC7941238

[7] Shabbir A , Cox A, Rodriguez-Menocal L, Salgado M, Badiavas EV. Mesenchymal stem cell exosomes induce proliferation and migration of normal and chronic wound fibroblasts, and enhance angiogenesis in vitro. Stem Cells Dev 2015;24:1635–47. 25867197 10.1089/scd.2014.0316 PMC4499790

[8] Fang S , Xu C, Zhang Y, et al. Umbilical cord-derived mesenchymal stem cell-derived exosomal MicroRNAs suppress myofibroblast differentiation by inhibiting the transforming growth factor-β/SMAD2 pathway during wound healing. Stem Cells Transl Med 2016;5:1425–39. 27388239 10.5966/sctm.2015-0367 PMC5031180

[9] Phinney DG , Pittenger MF. Concise review: MSC-derived exosomes for cell-free therapy. Stem Cells 2017;35:851–8. 28294454 10.1002/stem.2575

[10] Andreu Z , Yáñez-Mó1 MÃ. Tetraspanins in extracellular vesicle formation and function. Front Immunol 2014;5:442. 25278937 10.3389/fimmu.2014.00442 PMC4165315

[11] Zhang B , Yin Y, Lai RC, Tan SS, Choo AB, Lim SK. Mesenchymal stem cells secrete immunologically active exosomes. Stem Cells Dev 2014;23:1233–44 24367916 10.1089/scd.2013.0479

